# COVID-Washing in US Food and Beverage Marketing on Twitter: Content Analysis

**DOI:** 10.2196/37642

**Published:** 2022-10-14

**Authors:** Krystle A Tsai, Omni Cassidy, Josh Arshonsky, Sara Bond, Inés M Del Giudice, Marie A Bragg

**Affiliations:** 1 Department of Population Health New York University Grossman School of Medicine New York City, NY United States

**Keywords:** COVID-19, food and beverage marketing, Twitter, obesity, social media, nutrition, Twitter, risk factor, health advertising, marketing

## Abstract

**Background:**

Food companies have increased digital and social media ad expenditures during the COVID-19 pandemic, capitalizing on the coinciding increase in social media use during the pandemic. The extent of pandemic-related social media advertising and marketing tactics have been previously reported. No studies, however, have evaluated how food and beverage companies used COVID-washing on social media posts in the United States or analyzed the nutritional content of advertised food and beverage products. This study was designed to address these gaps by evaluating how food and beverage companies capitalize on the COVID-19 pandemic to promote unhealthy foods and sugary beverages.

**Objective:**

We aimed to document the types and frequencies of COVID-19–related themes in US food and beverage companies’ Twitter posts during the first wave of the pandemic in the United States, and assess the nutritional quality of food and beverage products featured in these tweets.

**Methods:**

Research assistants visited the Twitter accounts of the most-marketed food and beverage brands, and screen-captured all tweets posted between March 1 and May 31, 2020. Researchers documented the date of the tweet; the number of likes, views, comments, and “retweets”; and the type of food and beverage products. We coded tweets for the following 10 COVID-19 themes: (1) social distancing, staying home, or working remotely; (2) contactless delivery or pick-up; (3) handwashing or sanitizing; (4) masks; (5) safety or protection; (6) staying connected with others; (7) staying active; (8) frontline or essential workers; (9) monetary relief, donations, or unemployment; and (10) pandemic, unprecedented, or difficult times. Researchers calculated the nutrient profile index scores for featured foods and sorted beverages into categories based on sugar content.

**Results:**

Our final sample included 874 COVID-19–themed tweets from 52 food and beverage brands. Social distancing themes appeared most frequently (n=367, 42%), followed by pandemic, unprecedented, or difficult times (n=246, 28.2%), and contactless delivery (n=237, 27.1%). The majority of tweets (n=682, 78%) promoted foods and beverages. Among those tweets featuring foods and beverages, 89.6% (n=611) promoted unhealthy products, whereas 17.2% (n=117) promoted healthy products.

**Conclusions:**

Our findings point to a concerning marketing tactic in which major food and beverage companies promote unhealthy foods and sugary beverages during the COVID-19 pandemic. Given that nutrition-related diseases such as obesity and diabetes are risk factors for COVID-19 morbidity and mortality, food and beverage companies should reduce the promotion of unhealthy products to help decrease the prevalence of health conditions that place people at higher risk for severe illness and death due to COVID-19.

## Introduction

Health is determined not only by biology and individual choices but also by corporate practices and their influence on the social environments in which people live and work [1]. These “corporate determinants of health” were most recently defined as “strategies and approaches used by the private sector to promote products and choices that are detrimental to health” [2,3]. One example of a corporate determinant of poor health is unhealthy food and beverage marketing. Exposure to food advertising increases calorie consumption [4] and is particularly concerning during the current COVID-19 pandemic, given that obesity and diabetes are risk factors for COVID-19 morbidity and mortality [5-8].

Food marketing exerts a powerful influence on children’s and adults’ diets. To date, the majority of food advertising promotes products that are energy dense and nutrient poor [9]. Many research studies substantiate that such marketing of unhealthy foods affects young consumers’ preferences and purchasing behaviors, and increases their consumption of those marketed foods [4,9-14].

Social media use increased during the COVID-19 pandemic [15-19], coinciding with food and beverage companies’ growth in marketing efforts on social media. Companies that shifted their marketing dollars to digital media–centric platforms saw significant profit [20]: ad revenue from companies like YouTube, Facebook, and Twitter grew by up to 49% during the first quarter of 2021 [21]. The extent of pandemic-related social media advertising and marketing tactics have been previously reported [22-25]. Despite companies’ efforts to align with public health initiatives, COVID-washing may contribute to obesogenic environments and increase health risk. A content analysis of Australian-based social media posts from Australian food and beverage companies during the pandemic showed that 100% of unhealthy food and beverage parent companies included “COVID-washing” marketing techniques—the use of COVID-19–related themes (eg, social distancing) to market products [25]. The use of COVID-19–related marketing by food and beverage companies has also been documented in Latin America. A content analysis of Facebook posts of 5 fast food chains in Argentina, Bolivia, Guatemala, and Peru also documented the use of COVID-19–related marketing strategies during the pandemic [22], and a study in Brazil documented a high prevalence of unhealthy food advertising on the largest online food delivery platform in Latin America during the fourth month of the COVID-19 pandemic [23]. No studies, however, have evaluated how food and beverage companies used COVID-washing on social media posts in the United States or analyzed the nutritional content of advertised food and beverage products. Most of the world’s largest food companies are from the United States (eg, 5 of the top 10 largest food companies are from the United States) [26], and those US-based companies are increasingly targeting other countries with their unhealthy products [27], making it urgent to understand their domestic and international marketing practices. Conversely, Latin American brands do not have a large advertising presence in the United States—the most-marketed brands in the United States largely include US-based brands along with a few brands from outside of the United States (eg, Nestle based in Switzerland and Danone based in France) [26-30].

This study was designed to address these gaps by evaluating how food and beverage companies capitalize on the COVID-19 pandemic to promote unhealthy foods and sugary beverages. We aimed to document the types and frequencies of COVID-19–related themes in US food and beverage companies’ Twitter posts (ie, “tweets”), and assess the nutritional quality of featured products in these tweets.

## Methods

### Selection of Food and Beverage Brands

We conducted a content analysis of food and beverage brands’ US Twitter accounts during the first wave of the COVID-19 pandemic. We chose Twitter as the social media platform to analyze in our study because companies spend US $2.9 billion of their advertising budget on Twitter [31]. Additionally, our previous research shows that food and beverage brands use diverse and powerful advertising strategies on Twitter, including adopting personalities that drive consumer engagement and interaction on the platform [32]. To determine our sample of food and beverage brands, we selected a random subset of 29 of the most-marketed brands from the following categories, using the Rudd Center for Food Policy and Obesity’s Food Advertising to Children and Teens Scores (FACTS) reports: fast food [30], sugary drinks [33], cereals [34], and snacks [28]. Five research assistants then visited Twitter and searched for each brand’s official Twitter account page. On each account page, Twitter suggests three similar accounts in a “You might like” list. We also included these suggested accounts in our sample. Twitter uses an algorithm to populate these “You might like” lists, suggesting additional brands that are likely relevant and of interest to users who follow and interact with the initial brand account they are viewing. Our choice to include brands from these lists was because users who “follow” a particular brand account on Twitter are likely to also follow the suggested accounts in the “you might like” list on their account page. By also including these suggested brands, we aimed to produce a robust sample of popular food brands on Twitter. This search process yielded a sample of 52 brands—29 brands from the FACTS reports and 23 brands from Twitter’s “You might like” lists.

### Collection of Tweets From Brands’ Twitter Accounts

The lead author divided the 52 brands between seven researchers, who then visited their assigned brands’ Twitter accounts and recorded the number of “followers” each brand had. To collect our sample of tweets, the researchers then scrolled through each brand’s reverse-chronological feed of posted tweets, until they reached a tweet dated May 31, 2020. They then screen-captured each tweet posted between March 1 and May 31, 2020, and documented the following data for each captured tweet in an Excel (Microsoft Corporation) spreadsheet: date of posted tweet, number of likes, number of views, number of comments, and number of “retweets.” We only collected tweets and retweets generated by the brands, and did not collect any user-generated content. Finally, the researcher identified and documented each food and beverage product featured in the tweet. Products were included in the data collection if they were either referenced in the text of the tweet or depicted with an image of the product.

### Codebook Development and Qualitative Coding of Tweets

To create a codebook, the lead author and two senior researchers reviewed a subset of tweets in our sample to identify featured COVID-19–related themes. These identified themes were then documented and defined in a codebook to be used in a content analysis of the full sample of tweets. The lead author then trained the seven researchers on how to use the codebook to perform the qualitative coding. If throughout the coding process, a researcher identified an additional COVID-19–related theme, they notified the lead author who then updated the codebook. The final codebook included 10 distinct COVID-19–related themes: (1) social distancing, staying home, or working remotely; (2) contactless delivery or pick-up; (3) handwashing or sanitizing; (4) masks; (5) safety/protection; (6) staying connected with others; (7) staying active; (8) frontline or essential workers; (9) monetary relief, donations, or unemployment; and (10) pandemic, unprecedented, or difficult times (Table S1 in Multimedia Appendix 1).

Each tweet was then reviewed and coded for the 10 established COVID-19–related themes by one of the seven researchers. If there was a lack of clarity on how a tweet should be coded, the researcher raised the question with the research team, and through a team discussion, a consensus was reached. To account for coding consistency, the lead author reviewed 20% of the sample from each of the seven researchers. If a coding discrepancy was identified, the lead author discussed the discrepancy with the initial coder, and they reached a consensus. Researchers coded tweets for each theme that was featured, so some tweets were coded with more than one theme.

### Nutritional Analysis of Food and Beverages

We calculated nutrition scores for each featured food product using the validated Nutrient Profile Model (NPM) [35], with higher scores representing less healthy products and lower scores representing healthier products. We then converted the NPM scores to a more interpretable nutrient profile index (NPI) [34] score using the following formula: NPI score = −2 × NPM score + 70. NPI scores ranged from 1 to 100, with 1 being the worst nutrition score and 100 being the best score. We classified food items with an NPI score ≥64 as “healthy,” as this is the threshold for products that can be advertised to children in the United Kingdom [36]. The NPM is limited in its use with beverages and codes some sugary sweetened beverages as “healthy,” so we sorted featured beverages into the categories based on the sugar content outlined in the Rudd Center’s Sugary Drink FACTS Report [33]. Food and beverage products that were referenced in the text of the tweet or depicted with an image of the product were included in the nutritional analysis. In instances where a featured food or beverage product was not easily identifiable (eg, image of a brand’s take-out bag), we calculated and averaged the NPI scores of the brand’s top five marketed products, as described by Bragg et al [37].

## Results

**Figure 1:**
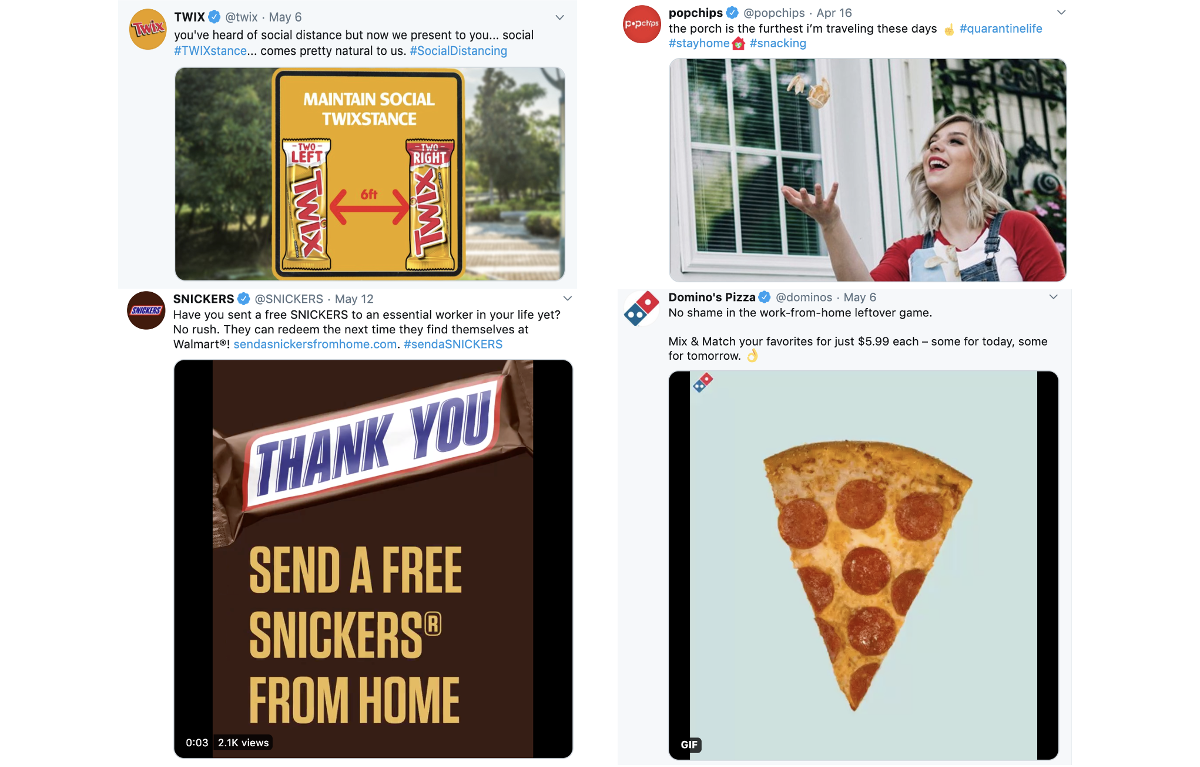
Four tweets in our sample that use COVID-19 themes (social distancing/staying home/working remotely; frontline/essential workers) to promote unhealthy food products.

After excluding brands without active Twitter accounts, we identified 52 unique food and beverage brands. During the study period, the 52 food and beverage brands collectively posted 2307 tweets, and 874 of those tweets featured COVID-19–related themes. Brands mentioned social distancing (n=367, 42% of COVID-19–themed tweets) most frequently in their tweets, followed by pandemic, unprecedented, or difficult times (n=246, 28.2%), contactless delivery (n=237, 27.1%), and frontline or essential workers (n=193, 22.1%; Figure 1). Half of the COVID-19–themed tweets (n=440, 50.1%) featured more than one COVID-19 theme. Engagement by followers (likes, comments, and retweets) varied between COVID-19 themes (Table 1) and brands (Table 2). Tweets featuring social distancing generated the most interactions (likes, comments, and retweets) of the 10 themes, with 118,838 likes; 11,268 comments; and 21,643 retweets (n=151,749, 23.2% of the total 655,551 interactions). Tweets referencing pandemic, unprecedented, or difficult times generated the next greatest number of interactions (n=124,632, 19% of the total interactions) with 96,423 likes; 6417 comments; and 21,792 retweets. Tweets referencing staying active generated the least number of interactions in our sample (n=1673, 0.3%).

Fast-food companies comprised more than one-quarter of the 52 brands in our sample (n=14, 27%) and generated more than half of the 874 COVID-19–themed tweets in our sample (n=461, 52.7%). Taco Bell (47/461, 10.2%), Little Caesars (46/461, 10%), and Chick-fil-A (41/461, 8.9%) posted the most COVID-19–themed tweets among fast-food brands, comprising 15.3% (134/874) of the total COVID-19–themed tweets in our sample. Fast-food companies had the majority of followers in our sample (27,274,600/37,063,870, 73.6%), and their tweets had the highest level of engagement, accruing the majority of the total number of “likes” (205,026/270,697, 75.7%), comments (20,333/27,633, 73.6%), and retweets (43,530/55,683, 78.2%). Contactless delivery was the most frequently referenced theme in the 461 tweets by fast-food brands (n=208, 45.1%), followed by social distancing (n=135, 29.3%), and pandemic (n=113, 24.5%).

Snack brands comprised over one-third of the 52 brands in our sample (n=19, 37%) and generated almost one-third of the 874 COVID-19–themed tweets in our sample (n=260, 29.7%). Snack brands had a total of 2,343,412 (6.3%) followers, and their tweets generated a total of 16,618 (6.1%) likes; 2454 (8.9%) comments; and 4187 (7.5%) retweets. Social distancing was the most frequently referenced theme in tweets by snack brands (132/260, 50.8%), followed by pandemic (93/260, 35.8%).

Beverage brands comprised 23% (n=12) of the 52 brands in our sample and had the second highest number of followers in our sample (n=7,104,432, 19.2%). Beverage brands generated 9.5% (n=83) of the COVID-19–themed tweets in our sample but had the second highest level of engagement, accruing a total of 47,691 (17.6%) likes; 4692 (17%) comments; and 7711 (13.9%) retweets. Social distancing was the most frequently referenced theme in the 83 beverage brand tweets (n=50, 60%), followed by staying connected (n=26, 31%) and pandemic (n=24, 29%).

A total of 78% (n=682) of the 874 tweets that featured COVID-19–related themes also promoted foods and beverages. Of these 682 COVID-19–related tweets, 89.6% (n=611) promoted unhealthy products, whereas 17.2% (n=117) promoted healthy products. Unhealthy foods were promoted more frequently (n=556, 81.5%) than sugary beverages (n=100, 14.7%). Healthy food and beverages were promoted in only 8.4% (n=57) and 9.4% (n=64) of tweets, respectively. A total of 10.4% (n=71) of tweets promoted only healthy products, whereas 82.8% (n=565) promoted only unhealthy products. A total of 38% (n=20) of the 52 brands in our sample promoted both healthy and unhealthy products in their COVID-19–themed tweets. Fast-food and snack brands promoted unhealthy products (n=367, 89.7% and n=168, 92.8%, respectively) more often than healthy products (n=59, 14.4% and n=24, 13.3%, respectively). Beverage brands promoted healthy products more often than unhealthy products (n=32, 62.8% vs n=27, 52.9%).

**Table 1:** Summary data for COVID-19–related themes featured across all tweets (N=874).

COVID-19 theme^a^	Frequency (N=1526), n (%)	Likes (N=503,649), n (%)	Comments (N=46,966), n (%)	Retweets (N=104,936), n (%)	Video views^b^ (N=32,065,091,897), n (%)
Social distancing, staying home, working remotely	367 (24.1)	118,838 (23.6)	11,268 (24.0)	21,643 (20.6)	25,378,029 (0.1)
Pandemic, unprecedented or difficult times	246 (16.1)	96,423 (19.1)	6417 (13.7)	21,792 (20.8)	16,000,164,700 (49.9)
Contactless delivery and pick-up	237 (15.5)	60,363 (12.0)	8802 (18.7)	15,085 (14.4)	3,996,010 (0.0)
Frontline and essential workers	193 (12.7)	63,689 (12.7)	5435 (11.8)	11,817 (11.3)	3,719,072 (0.0)
Monetary relief, donations, unemployment	192 (12.6)	37,860 (7.5)	3911 (8.3)	9260 (8.8)	16,005,367,147 (49.9)
Staying connected with others	125 (8.2)	63,414 (12.6)	4322 (9.2)	10,979 (10.5)	24,731,677 (0.0)
Safety, protection	74 (4.9)	37,531 (7.5)	4046 (8.6)	9077 (8.7)	128,128 (0.0)
Masks	51 (3.3)	10,271 (2.0)	794 (1.7)	1758 (1.7)	1,576,670 (0.0)
Handwashing, sanitizing	23 (1.5)	13,903 (2.8)	1894 (4.0)	3286 (3.1)	23,364 (0.0)
Staying active	18 (1.2)	1357 (0.3)	77 (0.2)	239 (0.2)	7100 (0.0)

^a^Some tweets included more than one theme, so the total number of theme instances is greater than the total number of tweets.

^b^Data only includes tweets that feature video media (n=192), as Twitter only reports the number of views for videos.

**Table 2:** Summary data for food and beverage brands in sample (N=52).

Brands	Tweets (N=874), n	Total followers (N=37,063,870), n	Total likes (N=270,697), n	Total comments (N=27,633), n	Total retweets (N=55,683), n	Total videos (N=167), n	Total video views (N=94,390,779), n	Category
3 Musketeers	17	3646	187	14	33	0	N/A^a^	Snacks
Altoids	3	1072	71	5	4	0	N/A	Snacks
Ben’s Original	3	7126	11	6	4	0	N/A	Other
Breyer’s	3	123,800	47	3	18	0	N/A	Snacks
Brisk Tea	2	34,700	18	3	4	1	270	Beverages
Cheerios	1	88,100	4	0	1	0	N/A	Cereal
Chick-fil-A	41	1,100,000	17,093	1316	2,818	7	111,200	Fast food
Chobani	15	103,300	633	29	160	1	16,900,000	Snacks
Cinnabon	30	169,600	2018	270	569	4	10,300	Fast food
Coca-Cola	9	3,300,000	3466	435	666	1	11,900	Beverages
Coffee Mate	1	59,400	67	8	8	0	N/A	Other
Combos	4	5712	84	12	22	0	N/A	Snacks
Domino’s	27	1,300,000	5448	1838	948	2	15,900	Fast food
Dunkin Donuts	39	1,200,000	10,851	802	1785	10	78,600	Fast food
Five Gum	2	22,200	13	7	2	1	261	Snacks
Gatorade	3	330,400	69	13	15	0	N/A	Beverages
Hellmann’s	5	36,400	51	4	18	2	695	Other
Kashi Foods	10	11,800	19	3	4	0	N/A	Snacks
Kellogg	63	77,300	1202	65	466	12	8256	Snacks
KFC	25	1,400,000	5306	1105	1165	9	106,800	Fast food
KitKat	11	409,300	995	43	207	5	10,700	Snacks
Kool-Aid	6	74,200	26,112	1122	4070	1	961,700	Beverages
Krispy Kreme	22	346,000	14,492	477	3070	1	5500	Fast food
Lay’s	6	457,500	606	254	173	2	2461	Snacks
Lipton Tea	4	55,000	135	5	33	0	N/A	Beverages
Little Caesars	46	316,200	9917	915	2679	22	5,830,600	Fast food
Lunchables	4	9982	70	21	19	0	N/A	Snacks
M&Ms	9	118,400	811	50	135	2	2192	Snacks
Magnum Ice Cream	4	56,500	53	14	6	2	932	Snacks
McDonald’s	22	3,600,000	23,946	3430	3098	2	159,700	Fast food
Miracle Whip	4	17,100	37	16	6	0	N/A	Other
Oscar Meyer	35	38,100	2243	192	631	6	74,342	Snacks
Panera Bread	39	460,800	8249	632	1797	9	1,534,900	Fast food
Pepsi	9	3,000,000	15,607	2857	2567	2	68,091,000	Beverages
Perrier USA	6	14,300	28	1	9	1	149	Beverages
Pizza Hut	38	1,600,000	4022	1127	923	17	94,000	Fast Food
Poland Spring	7	7932	56	2	13	3	397	Beverages
Popchips	38	48,800	89	17	46	10	1529	Snacks
Skittles	7	407,000	6505	1340	1519	2	116,800	Snacks
Snickers	10	373,100	2207	245	643	3	6300	Snacks
Sonic Drive-In	32	382,000	2608	451	603	10	27,900	Fast food
Starbucks	34	11,100,000	66,380	4360	13,381	3	101,800	Fast food
Subway	19	2,300,000	2014	391	513	2	8700	Fast food
Taco Bell	47	1,900,000	32,682	3219	10,181	3	101,800	Fast food
TGI Fridays	26	214,200	464	57	135	6	5100	Other
Topo Chico	10	10,600	107	9	22	0	N/A	Beverages
Tostitos	7	40,000	128	16	19	0	N/A	Snacks
Tropicana	1	185,800	9	1	2	0	N/A	Beverages
Twix	12	34,900	655	122	80	1	898	Snacks
Velveeta	30	20,100	728	65	83	0	N/A	Other
Vita Coco	15	57,900	2060	243	304	1	7100	Beverages
Zico Coconut Water	11	33,600	24	1	6	1	97	Beverages

^a^N/A: not applicable.

## Discussion

### Principal Findings

We identified a wide range of COVID-19–related themes. Brands used social distancing themes most frequently, often with a play-on-words using the brand name or slogan (eg, “Maintain Social TWIXstance” or “Lunchables from home”). During the first wave of the COVID-19 pandemic, between March and May 2020, we found that 78% (n=682) of our sampled tweets with COVID-19–related themes promoted food and beverage products, of which the majority were unhealthy.

Our findings are consistent with previous studies that have analyzed food and beverage marketing during the COVID-19 pandemic, showing that major food and beverage brands worldwide frequently posted on social media using COVID-19–related themes [25,38,39]. In our sample of tweets, companies most frequently referenced social distancing themes (eg, “OH____6 ft____YEAH!” [Kool-Aid], “No shame in the work-from-home leftover game” [Domino’s Pizza], “having your breaks at a distance” [KitKat], or “Quarantine Cuisine” [Popchips]). This finding is consistent with previous studies that examined other social media platforms like Facebook and Instagram where the majority of posts from major food and beverage brands referenced “isolation activities” (eg, #stayhome) [25]—activities that are included under our “social distancing/staying home/working remotely” theme. Discussing social distancing and isolation activities may be useful for food and beverage brands. With millions of families remaining in their homes, people have turned to the internet to order food and beverages. The four major US food delivery apps—Grubhub, DoorDash, Uber Eats, and Postmates—reported a collective US $3 billion increase in revenues in the second and third quarters of 2020 due to the pandemic’s shelter-in-place restrictions [40]. Particularly concerning is the increase in online ordering of fast food that continued to increase through 2021. A recent report by UpMenu, an online food ordering software service provider, reported that Chick-fil-A experienced a 590% increase in online ordering during the pandemic, followed closely behind by Burger King and Little Caesars with increases of 407% and 279%, respectively [40,41]. Our findings that Little Caesars and Chick-fil-A were in the top 4 most active brands posting COVID-19–themed content in our sample, suggests that COVID-washing may have played a role in this increase in online ordering during the pandemic.

Using the NPM, this study is the first to also examine the nutritional quality of products promoted in companies’ COVID-19–related tweets. The analysis revealed that most products were classified as unhealthy, which is consistent with other analyses of food and beverage advertisements [42-47]. However, COVID-19 raises new ethical considerations for food and beverage companies’ marketing practices because diet-related diseases like obesity and diabetes are major risk factors for severe illness and death due to COVID-19.

These findings point to a concerning yet typical marketing tactic of food and beverage companies. Corporate marketers taking advantage of trending social or health issues for profit is a textbook strategy from the corporate marketing playbook. However, using the uncertainty and disruptions of the COVID-19 pandemic for promotion is particularly more egregious than say seasonal or holiday food marketing campaigns because overconsuming unhealthy food and drink can indirectly increase the risk of more severe COVID-19–related outcomes.

Posting on Twitter and other social media platforms has enabled companies to market themselves to a wide audience, as most of the US population during our study period followed stay-at-home orders and spent more time online than ever before. Scholars refer to this kind of corporate activity as a type of cause marketing or corporate social responsibility [48] strategy, whereby companies align themselves with certain social or health issues to enhance their own image. COVID-washing portrays food and beverage companies as empathetic and responsive to the pandemic. In reality, however, this is a classic corporate strategy that may contribute to poor diet and exacerbate poor outcomes related to COVID-19.

### Limitations

This study has several limitations. One limitation is that it is possible we did not capture every food and beverage brand’s Twitter account in our sample. We included a subset of the top marketed brands from the Rudd Center for Food Policy and Obesity’s FACTS reports in our sample, which likely omitted smaller food and beverage brands. While we also included the additional brands suggested in Twitter’s “You might like” list on each brands’ Twitter account, these suggested lists were not comprehensive, as they only include three brands each. It is therefore possible that there are additional food and beverage brands that we did not capture in our sample. Further, as only Twitter was included in our sample, we only captured a subset of these food and beverage brands’ COVID-19–themed marketing. It is possible that some food and beverage companies may maintain a more active marketing presence on other social media platforms such as Instagram, TikTok, and Facebook. Future studies should examine COVID-19–themed marketing across all social media platforms.

A second potential limitation is that we selected the brands in our sample from unhealthier food categories (fast food, sugary drinks, snacks, and cereal) and did not focus on brands in healthier categories. Our sample did, however, include some brands with healthy products (eg, Perrier, Poland Spring, Chobani, or Subway), and many of the unhealthy brands in our sample also offer some items that are healthy. Over one-third of the brands (n=20, 39%) in our sample featured a mix of healthy and unhealthy products in their tweets; however, the healthier options were not products that appeared as frequently in the ads in our sample. Future research could examine the prevalence of COVID-washing by healthier food and beverage brands; however, we predict this would likely comprise a tiny proportion of the overall food marketing landscape because we know from previous research that the unhealthy brands spend more on advertising than healthy brands [28,30,33] and that food companies mostly promote their unhealthy products on their social media accounts [49-51]. Because of this discrepancy, we were less interested in comparing healthy versus unhealthy brands and more interested in documenting the extent to which these unhealthy brands are promoting junk food. This phenomenon is problematic because unhealthy brands have the most money to spend and should not be capitalizing on the pandemic to sell products given the risk factors of obesity and diabetes related to COVID-19.

Another limitation is that we were unable to measure the actual exposure and impressions of these Twitter posts. Finally, we did not examine how these COVID-19–themed ads may directly affect the dietary and purchasing choices of consumers, which is an area of future research.

### Conclusions

Our findings document the prevalence of food and beverage companies’ use of COVID-19 themes in tweets that promote unhealthy foods and sugary beverages. Given that obesity and diabetes are risk factors for COVID-19 morbidity and mortality, food and beverage companies should reduce the promotion of unhealthy products to help decrease the prevalence of health conditions that place people at higher risk for severe illness and death due to COVID-19.

## Appendix

Multimedia Appendix 1 Table S1.

## Abbreviations

FACTS: Food Advertising to Children and Teens Scores
NPI: nutrient profile index
NPM: Nutrient Profile Model
