# Supplementary material for: COVID-Washing in US Food and Beverage Marketing on Twitter: Content Analysis
Source: JMIR Form Res. 2022 Oct 14;6(10):e37642. doi: 10.2196/37642 (PMC9578516; doi:10.2196/37642)
Supplement: Multimedia Appendix 1 [file formative_v6i10e37642_app1.docx]

**TABLE S1.** COVID-related marketing themes in food and beverage brands’ tweets

| **COVID-19 Theme** | **Description** | **Example** |
| --- | --- | --- |
| Social distancing/staying home/working remotely | Mentions social distancing, including staying home and/or working remotely; brand suggestions for things to do while staying home | *We think these stories are a *little* funny, but #letsbeblunt: isn’t today supposed to be about being chill? Tell us how it really is staying home with your little ones using the hashtag, and we’ll send you a free case of Vita Coco Infused with Hemp to help you…escape. (Twitter, @VitaCoco)* |
| Contactless delivery/pick-up | Mentions of contactless delivery or pick-up options during the COVID-19 pandemic | *#TheGreatAmericanTakeout is back! Support local restaurants and get your favorite meals to go! Use the SONIC App for contactless ordering and payment. Summer’s here, and with it the Red Bull Summer Edition Slush! Try it for ½ Price during Happy Hour only on the SONIC app! (Twitter, @sonicdrivein)* |
| Hand-washing/sanitizing | References hand-washing or sanitizing to reduce spread of COVID-19 during food handling and/or delivery | *Safety is our #1 priority: (1) Stores increased their already frequent sanitation of high-contact areas (2) Hands are washed frequently, before & after each delivery (3)Your pizza remains contact-free after it leaves our 450F oven. (Twitter, @dominos)* |
| Masks | Mentions masks in some capacity | *Shout out to Crystal Ford and her daughter of Evansville, IN, for making these amazing handcrafted face masks for her crew. #ProudtoServe (Twitter, @pizzahut)* |
| Safety/protection | Ensuring safety and protection among customers and employees | *From our CEO regarding COVID-19:*  *“I want to assure you that the safety and well-being of customers, employees, and franchises is our top priority.” (Twitter, @littlecaesars)* |
| Staying connected with others | References to staying connected with family or friends or colleagues | *What better way to stay connected with friends and colleagues than over a meal? Learn how Kellogg Culinary Director Mark Graham is bringing #TeamKellog together with live cooking demos broadcast from his Chicago apartment. (Twitter, @KelloggCompany* |
| Staying active | Promoting physical activity with reference to COVID-19 and/or sharing workout/training advice | *Wonder how a tennis icon makes the days count? @SerenaWilliams is sharing an exclusive peek into her at-home training routine with a lower-body circuit that was designed to keep you strong & ready to get back on the court. #makethedayscount (Twitter, @Gatorade)* |
| Frontline/essential workers | Referencing or thanking essential workers and health workers | *Friends. Family. Community. WE NEED YOU! Please help us help our frontline workers. We want to give them a FREE thank you meal. If you know anyone who could use one, please like, tag in comment or retweet to let them know. We’d truly be proud to take their order. (Twitter, @McDonalds)* |
| Monetary relief/donations/unemployment | References large product or monetary donations | *Starbucks remains committed to supporting the health and well-being of our partners (employees) and their families. Today, we are announcing a $10 million global relief fund dedicated to supporting partners affected by the COVID-19 outbreak. (Twitter, @Starbucks)* |
| Pandemic/unprecedented/difficult times | References to the pandemic and the unprecedented, or unexpected, or difficult time emerging from it | *We are grateful for the guests and communities we serve. That’s why we are now offering FREE DELIVERY now through 3/31/20. Because you deserve good, clean food during these unprecedented times. Stay safe, stay healthy, stay well. Order now (Twitter, @panerabread)* |
